# Cognitive Factors Associated With Public Acceptance of COVID-19 Nonpharmaceutical Prevention Measures: Cross-sectional Study

**DOI:** 10.2196/32859

**Published:** 2022-05-13

**Authors:** Aymery Constant, Donaldson Conserve, Karine Gallopel-Morvan, Jocelyn Raude

**Affiliations:** 1 Ecole des Hautes Etudes en Santé Publique Rennes France; 2 Department of Prevention and Community Health Milken Institute School of Public Health George Washington University Washington, DC United States

**Keywords:** Extended Parallel Process Model, COVID-19, lockdown, public acceptance, nonpharmaceutical measures, Likert scale, France

## Abstract

**Background:**

During the COVID-19 crisis, protests against restrictions emerged and rule violations increased, provoking peaks in new positive cases, forcing authorities in France to impose fines to slow down the spread of the disease. Due to these challenges, subsequent implementations of preventive measures in response to COVID-19 recurrences or other pandemics could present difficulties for decision makers. A better understanding of the factors underlying the public acceptance of COVID-19 nonpharmaceutical preventive measures may therefore contribute greatly to the design of more effective public communication during future pandemics.

**Objective:**

The aim of this study was to evaluate the acceptance of COVID-19 nonpharmaceutical prevention measures in France. The specific objectives were (1) to examine the public’s acceptance of COVID-19 nonpharmaceutical prevention measures and (2) to assess the association of the public’s acceptance of these prevention measures and their perception of COVID-19.

**Methods:**

Data were collected from 2004 individuals through an online survey conducted 6-8 weeks after the first lockdown in France. For objective 1, participants were asked the extent to which they supported 8 COVID-19 nonpharmaceutical preventive measures using a 4-point Likert scale. For objective 2, COVID-19–related perceptions were assessed using a 5-point Likert scale from an adapted version of Witte’s Extended Parallel Process Model. Sociodemographic and environmental variables were also collected. The public’s acceptance factors were estimated using an unweighted least squares factorial analysis, and their associations with perceptions of COVID-19, expressed as rate ratios (RR) and 95% CIs, were estimated using generalized linear Poisson regression models. Statistical analyses were performed using the SPSS statistical package.

**Results:**

The acceptance rate reached 86.1% for individual protective measures, such as making masks mandatory in public open spaces, and 70.0% for collective restrictions, such as isolating the most vulnerable people (1604/2004, 80%) or forbidding public gatherings (n=1590, 79.3%). The least popular restrictions were closing all schools/universities and nonessential commerce such as bars and restaurants (n=1146, 57.2%). Acceptance of collective restrictions was positively associated with their perceived efficacy (RR 1.02, 95% CI 1.01-1.03), fear of COVID-19 (RR 1.04, 95% CI 1.03-1.05), and perceived severity of COVID-19 (RR 1.04, 95% CI 1.03-1.06), and negatively with age >60 years (RR 0.89, 95% CI 0.81-0.98). Acceptance of individual protective measures was associated with their perceived efficacy (RR 1.03, 95% CI 1.03-1.04), fear of COVID-19 (RR 1.02, 1.01-1.03), and perceived severity of COVID-19 (RR 1.03, 1.01-1.05).

**Conclusions:**

Acceptance rates of COVID-19 nonpharmaceutical measures were rather high, but varied according to their perceived social cost, and were more related to collective than personal protection. Nonpharmaceutical measures that minimize social costs while controlling the spread of the disease are more likely to be accepted during pandemics.

## Introduction

### Background

The COVID-19 pandemic has affected many countries, with more than 10 million cases worldwide and more than 500,000 deaths as of July 1, 2020 [1]. Several restrictions were implemented to prevent further spread of the disease in the early stages of the pandemic. Confinement, the restriction of individuals to their homes, was one of the restrictions enforced in many countries [2], including France beginning on March 17, where surveillance of COVID-19 cases was implemented on January 10, 2020 [3]. In addition, global and local health authorities used media campaigns to inform individuals about the spread of the virus, the number of daily cases and deaths, and recommended actions to prevent infections [4,5]. The preventive measures include regular handwashing, social distancing, avoiding crowded places, and covering the mouth and nose, among others.

The lockdown was lifted in France on May 11, 2020, after a dramatic decrease in the number of cases and deaths, but mobility restrictions had some major adverse consequences [6]. The ensuing reductions in social (collective training sessions or sport events) and physical (barred access to exercise facilities or parks) opportunities to exercise had a direct negative effect on health behaviors and well-being [7-11]. The lockdown also had a detrimental impact on various aspects of psychological health (eg, posttraumatic stress disorder, anxiety, and depression [12,13]), especially in high-density and socially deprived neighborhoods [14] and among people with pre-existing chronic conditions [15]. Social distancing, self-isolation, and travel restrictions have led to a reduced workforce across all economic sectors and caused many jobs to be lost. Schools were closed and the need for commodities and manufactured products decreased [16]. As a result, protests against restrictions emerged and rule violations increased, provoking peaks in new positive cases [17], forcing authorities to impose fines to slow down the spread of COVID-19 [18]. Due to these challenges, subsequent implementations of nonpharmaceutical measures in response to COVID-19 recurrences or other pandemics could present difficulties for decision makers [19]. A study examining acceptance of different scenarios showed that lockdown length affected respondents’ reactions much more strongly than intensity or flexibility [20]. Additional analyses showed that half of the respondents rejected any further extensions or intensifications, while 20% would endorse long-term strategies if necessary.

### Study Rationale

Beliefs and risk perceptions associated with the disease (perceived personal vulnerability and perceived severity of the disease) have a major influence on the acceptance and uptake of and adherence to required restrictions [21-26]. This study was based on the Extended Parallel Process Model (EPPM). During the first lockdown in France, we investigated COVID-19 fear, risk perception, and trust in recommended measures based on the EPPM [27], which is one of the latest developments among theories that explain the role of fear in persuasion. The following constructs are central to the EPPM: fear, threat (with its two components: perceived severity of and perceived susceptibility to the illness), efficacy (comprising self-efficacy and response efficacy), and two types of responses (danger control and fear control). As nonpharmaceutical interventions play a considerable role in the control and prevention of pandemics such as the COVID-19 pandemic, it is necessary to better understand the factors underlying their public acceptance.

### Specific Objectives

The objectives of this study were (1) to measure the public’s acceptance of COVID-19 nonpharmaceutical measures and (2) to assess the association of the public’s acceptance of these measures and their perception of COVID-19.

## Methods

### Study Design

Data were collected from a 2-week cross-sectional survey administered 6-8 weeks after the first lockdown (June 25-July 5, 2020) among adults residing in France.

### Participants and Procedures

The respondents were recruited among Arcade Research panelists, who agreed to participate regularly in surveys of customer attitudes and experiences. The respondents to this survey were enrolled on the basis of a stratified sampling method to reflect the distribution of the French general population regarding sex, age, occupation, and region.

### Ethical Considerations

The research protocol was registered by the École des Hautes Études en Santé Publique (EHESP) School of Public Health Office for Personal Data Protections and approved by the Institutional Review Board of the Méditerranée Infection University Hospital Institute (reference number: 2020-022).

### Measurements

#### Acceptance of Public COVID-19 Nonpharmaceutical Measures

The dependent variable for the analyses was support of the following eight restrictive measures implemented (or likely to be implemented) by national governments to contain the COVID-19 outbreak: (1) make face masks mandatory in public closed spaces; (2) make face masks mandatory in public open spaces; (3) isolate vulnerable people (eg, older adults); (4) forbid public gatherings (eg, fairs, markets); (5) implement mobility restrictions for nonessential workers; (6) introduce a stay-at-home order for nonessential workers; (7) close all schools/universities; and (8) close nonessential commerce (eg, bars, restaurants). For each of them, the participants were asked to rate their acceptance on a Likert-type response scale, which ranged from 1 (“totally disagree”) to 4 (“totally agree”), and for which the meaning of each value was explicitly indicated [28]. To facilitate the treatment of the data, agreements obtained from these 8 items were added to generate a cumulative score that enabled the research team to assess participants’ acceptance of proposed nonpharmaceutical measures.

#### Sociocognitive Factors

To assess participants’ beliefs and expectations related to the COVID-19 epidemic, we used a range of constructs and variables from Witte’s EPPM. Items related to these constructs were adapted to the COVID-19 pandemic and translated into French. EPPM factors were estimated using an unweighted least squares factorial analysis, followed by a Promax rotation, and five factors were extracted accordingly [8]: (1) efficacy of preventive measures (eg, *actions recommended by scientists are effective at preventing COVID-19*), (2) lack of fear control (eg, *the risk of being infected is frightening me*), (3) perceived severity of COVID-19 (eg, *I believe that COVID-19 is extremely harmful*), (4) perceived susceptibility to COVID-19 (eg, *it is possible that I will get COVID-19 in the next few weeks*), and (5) cognitive avoidance (eg, *When I go shopping, I tend to avoid thinking about the risk of being infected*).

Sociodemographic and environmental variables were also collected, such as age in years (divided into groups: 18-39 years, 40-59 years, and ≥60 years), gender (self-reported sex), occupational status (active, unemployed, or retired), persons in household (≥3, 2, or 1), living density (urban, more than 100,000 people; urban, 20,000-100,000 people; urban, 2000-20,000 people; rural), chronic disease (yes/no), and perceived health (very poor, poor, good, very good).

### Data Analysis

Categorical data were expressed as frequencies (n) and percentages (%), while numerical data were expressed as mean (SD), and compared with 1-way ANOVA. EPPM raw scale scores were transformed to a 0-100 scale: ([raw score – lowest possible raw score]/possible raw score range) × 100. Acceptance factors were estimated using an unweighted least squares factorial analysis, followed by a Promax rotation, a nonorthogonal (oblique) solution in which the factors are allowed to be correlated. This method provides accurate and conservative parameter estimates when using ordinal data [29]. This item reduction method established which of the 8 items belonged to domains or conceptual areas and which items should be maintained. Items are deleted if they loaded on 2 or more factors, or if they exhibited a correlation coefficient of less than 0.40 with their own factor. Internal consistency reliability was assessed by computing Cronbach α, considered satisfactory if ≥.70 [30]. Interscale correlations were computed with the nonparametric Spearman correlation test. Since the study outcomes were count variables (number of accepted measures), generalized linear Poisson regression models were used to estimate the rate ratios (RRs) of acceptance as a function of sociodemographic variables and scores of COVID-19 perceptions, as assessed by the EPPM. Estimates in univariate analysis (model 1) were expressed as RRs with 95% CIs. Significant estimates from model 1 were analyzed in a multivariate model (model 2). The goodness of fit of the multivariate model was assessed using the value/df for the deviance statistics. This value should be near 1.0 for a Poisson regression. Statistical analyses were performed using the SPSS statistical package (version 19; IBM Corp).

## Results

### Participant Characteristics

Of the 2004 individuals who completed the survey (Table 1), half were women (1012/2004, 50.5%), 66% (1329/2004) were professionally active, and 76% (1532/2004) were living in urban environments. The mean age was 46.9 (SD 15.9) years, and was similar between men (mean 46.4, SD 16.3) and women (mean 47.4, SD 15.5; *P*=.18)

More than 1 in 5 participants (404/2004, 20.5%) reported financial difficulties related to COVID-19, and 3 in 10 had a chronic disease (n=615, 30.7%). Nearly 9 in 10 respondents (n=1796, 89.6%) perceived their health state as “good” or “very good.”

**Table 1:** Participants’ characteristics (N=2004).

Variables			Values
**Gender, n (%)**			
	Male	992 (49.5)	
	Female	1012 (50.5)	
**Age group (years)** **, n (%)**			
	≥60	518 (25.8)	
	40-59	750 (37.1)	
	18-39	736 (36.7)	
**Professional status** **, n (%)**			
	Active	1329 (66.3)	
	Retired	427 (21.3)	
	Unemployed	248 (12.4)	
**People in the household** **, n (%)**			
	≥3	825 (41.2)	
	2	723 (36.1)	
	1	456 (22.8)	
**Population density** **, n (%)**			
	Urban, more than 100,000 people	385 (19.2)	
	Urban, 20,000-100,000 people	520 (25.9)	
	Urban, 2000-20,000 people	627 (31.3)	
	Rural zone	472 (23.6)	
Chronic disease, n (%)			615 (30.7)
**Perceived health** **, n (%)**			
	Poor/very poor	208 (10.4)	
	Good/very good	1796 (89.6)	
**Financial difficulties** **, n (%)**			
	Yes, related to COVID-19	404 (20.2)	
	Yes, unrelated to COVID-19	480 (24)	
	None	1120 (55.9)	
**EPPM^a^ scores, mean (SD)**			
	Efficacy	73.8 (17.4)	
	Fear control	54.5 (26)	
	Severity	73.5 (23.1)	
	Vulnerability	42.7 (22.4)	
	Avoidance	48.9 (22.9)	

^a^EPPM: Extended Parallel Process Model.

### Public Acceptance of Nonpharmaceutical COVID-19 Measures

The majority of the study population approved of all 8 proposed measures (Table 2). The items with the highest approval ratings were “make masking mandatory in public closed spaces” (1783/2004, 89.0%) and “make masking mandatory in public open spaces” (n=1667, 83.2%), and the items with the lowest approval ratings were “closing all schools/universities” (n=1286, 64.2%) and “closing nonessential commerce such as bars and restaurant (n=1146, 57.2%).

Unweighted least squares exploratory factorial analysis, followed by a Promax rotation, was performed on the 8 items. Eigenvalues for the first 3 factors were 4.58, 1.05, and 0.63, respectively; this suggested a 2-factor solution explaining 62.5% of the common variance of the data. Factor 1 included 6 items related to collective restrictions and was interpreted as expressing acceptance of collective restrictions, whereas factor 2 included the 2 items related to mandatory mask wearing and was interpreted as expressing acceptance of individual protective measures. The factors showed satisfactory internal validity (Cronbach α was 0.88 for factor 1 and 0.87 for factor 2). The interscale correlation coefficient (*r*=0.61) showed that these factors were related but distinct. On average, more than 80% of the study population agreed with individual protective measures (make masking mandatory in public closed spaces: 1783/2004, 89%; make masking mandatory in public open spaces: n=1667, 83.2%) and 74% agreed with collective restrictions, with some variations—from 80% (n=1604) for “isolate vulnerable people” to 57.2% (n=1146) for “close nonessential commerce such as bars and restaurants.” More than 80% (n=1628) of participants accepted the 2 proposed individual protective measures and 9.1% (n=182) rejected them both, while 41.1% (n=823) accepted the 6 proposed collective restrictions and 6.1% (n=122) rejected all of them (Table 3).

Regarding COVID-19 perceptions, as assessed by the EPPM, efficacy (mean 73.8, SD 17.4) and severity (mean 73.5.1, 23.1) had the highest scores on a 100-point response scale, followed by lack of fear control (mean 54.5, SD 26.0), cognitive avoidance (mean 48.8, SD 22.9), and perceived vulnerability (mean 42.8, SD 22.4). Differences between T-scores were significant, except for efficacy and severity.

**Table 2:** Numbers, percentages, and factor loadings for the 2-factor solution of the acceptance of 8 nonpharmaceutical COVID-19 measures (N=2004).

Item	Totally agree/agree, n (%)	Totally disagree/disagree, n (%)	Factors	
			F1	F2
Make mask mandatory in public closed spaces	1783 (89)	221 (11)	N/A^a^	0.95
Make mask mandatory in public open spaces	1667 (83.2)	337 (16.8)	N/A	0.81
Isolate vulnerable people (eg, older adults)	1604 (80)	400 (20)	0.56	N/A
Forbid mass gatherings (eg, fairs, markets)	1590 (79.3)	414 (20.7)	0.59	N/A
Mobility restrictions for nonessential workers	1482 (74)	522 (26)	0.74	N/A
Stay at home order for nonessential workers	1314 (65.6)	690 (34.4)	0.85	N/A
Close all schools/universities	1286 (64.2)	718 (35.8)	0.80	N/A
Close nonessential commerce (eg, bar, restaurant)	1146 (57.2)	858 (42.8)	0.82	N/A
Eigenvalue	N/A	N/A	4.58	1.05
Percentage of explained variance	N/A	N/A	52.6	9.9
Cronbach α	N/A	N/A	0.88	0.87

^a^N/A: not applicable.

**Table 3:** Respondents (N=2004) agreeing with proposed collective COVID-19 nonpharmaceutical prevention measures.

Number of measures accepted	Respondents, n (%)
0	122 (6.1)
1	149 (7.4)
2	186 (9.3)
3	209 (10.4)
4	239 (11.9)
5	276 (13.8)
6	823 (41.1)

### Association Between Public’s Acceptance of Nonpharmaceutical Measures and COVID-19 Perceptions

Estimate of acceptance of collective restrictions in univariate analysis (Table 4) increased with household number and level of efficacy, fear, perceived severity, perceived susceptibility, and cognitive avoidance and decreased with age older than 60 years and retired occupational status. In multivariate analyses, this estimate increased with elevated level of efficacy, fear, and perceived severity and decreased with age older than 60 years.

Estimate of acceptance of individual protective measures in univariate analysis (Table 5) increased with level of efficacy, fear, perceived severity, and perceived susceptibility. In multivariate analyses, this estimate increased with higher level of efficacy, fear, and perceived severity. However, the goodness of fit for the multivariate model indicated an underdispersion of the data that warrants caution when interpreting the results.

**Table 4:** Rate ratios and 95% CIs of the acceptance of collective restrictions (N=2004), Poisson regression.^a^

Variables			Univariate, rate ratio (95% CI)		Multivariate^b^, rate ratio (95% CI)
**Gender**					
	Female	1.03 (0.98-1.07)		N/A^c^	
	Male	1		N/A	
**Age in years**					
	≥60	*0.89 (0.84-0.94)*		*0.89 (0.81-0.98)*	
	40-59	0.97 (0.92-1.02)		0.96 (0.91-1.01)	
	18-39	1		1	
**Professional status**					
	Active	1.01 (0.94-1.07)		1.02 (0.96-1.09)	
	Retired	*0.91 (0.85-0.99)*		0.98 (0.88-1.09)	
	Unemployed	1		1	
**Population density**					
	Urban, more than 100,000	1.00 (0.94-1.07)		N/A	
	Urban, 20,000-100,000	1.04 (0.98-1.10)		N/A	
	Urban, 2000-20,000	1.04 (0.98-1.10)		N/A	
	Rural zone	1		N/A	
**Household size**					
	≥3	*1.11 (1.05-1.18)*		1.04 (0.99-1.11)	
	2	1.03 (0.97-1.09)		1.03 (0.97-1.09)	
	1	1		1	
Chronic disease			1.00 (0.95-1.05)		N/A
**Perceived health**					
	Poor/very poor	0.96 (0.89-1.03)		N/A	
	Good/very good	1		N/A	
**Financial difficulties**					
	Yes, related to covid	1.07 (1.02-1.13)		N/A	
	Yes, unrelated to covid	1.01 (0.96-1.07)		N/A	
	None	1		N/A	
**EPPM^d^ scores**					
	Efficacy	*1.03 (1.02-1.04)*		*1.02 (1.01-1.03)*	
	Lack of fear control	*1.06 (1.05-1.07)*		*1.04 (1.03-1.05)*	
	Severity	*1.08 (1.07-1.09)*		*1.04 (1.03-1.06)*	
	Vulnerability	*1.05 (1.04-1.06)*		1.01 (0.99-1.02)	
	Avoidance	*1.02 (1.01-1.03)*		1.00 (0.99-1.02)	

^a^Significant results (*P*<.05) are marked in italics.

^b^Goodness of fit for the multivariate model (value/df for the deviance)=1.08.

^c^N/A: not applicable.

^d^EPPM: Extended Parallel Process Model.

**Table 5:** Rate ratios and 95% CIs of the acceptance of individual protective measures (N=2004), Poisson regression.^a^

Variables			Univariate, rate ratio (95% CI)		Multivariate^b^, rate ratio (95% CI)
**Gender**					
	Female	1.04 (0.97-1.11)		N/A^c^	
	Male	1		N/A	
**Age group (years)**					
	≥60	1.08 (0.99-1.17)		N/A	
	40-59	1.04 (0.96-1.12)		N/A	
	18-39	1		N/A	
**Professional status**					
	Active	1.02 (0.92-1.13)		N/A	
	Retired	1.09 (0.97-1.23)		N/A	
	Unemployed	1		N/A	
**Population density**					
	Urban, more than 100,000 people	0.95 (0.86-1.06)		N/A	
	Urban, 20,000-100,000 people	0.99 (0.90-1.09)		N/A	
	Urban, 2000-20,000 people	1.01 (0.92-1.10)		N/A	
	Rural zone	1		N/A	
**Number of household**					
	≥3	1.04 (0.95-1.14)		N/A	
	2	1.04 (0.95-1.14)		N/A	
	1	1		N/A	
Chronic disease			1.07 (0.99-1.15)		N/A
**Perceived health**					
	Poor/very poor	0.96 (0.86-1.07)		N/A	
	Good/very good	1		N/A	
**Financial difficulties**					
	Yes, related to covid	0.98 (0.90-1.07)		N/A	
	Yes, unrelated to covid	1.01 (0.963-1.09)		N/A	
	None	1		N/A	
**EPPM^d^ scores**					
	Efficacy	*1.04 (1.03-1.05)*		*1.03 (1.03-1.04)*	
	Lack of fear control	*1.04 (1.03-1.05)*		*1.02 (1.01-1.03)*	
	Severity	*1.07 (1.05-1.08)*		*1.03 (1.01-1.05)*	
	Vulnerability	*1.03 (1.01-1.04)*		1.00 (0.98-1.02)	
	Avoidance	1.00 (0.98-1.02)		N/A	

^a^Significant results are marked in italics.

^b^Goodness of fit for the multivariate model (value/df for the deviance)=0.34.

^c^N/A: not applicable.

^d^EPPM: Extended Parallel Process Model.

## Discussion

### Principal Results

Acceptance rates in our study population reached, on average, 86.1% for individual protective measures (such as mandatory face mask wearing), and 74.0% for collective restrictions, such as isolate vulnerable people (80%), forbid public gatherings (79.3%), and mobility restrictions for nonessential workers (74.0%). The least popular restrictions were closing of nonessential commerce such as bars and restaurants (57.2%). Acceptance of collective restrictions was positively associated with the level of efficacy, fear, and perceived severity, and negatively with age older than 60 years. Acceptance of individual protective measures was associated with level of efficacy, fear, and perceived severity.

Data were collected after the first lockdown in France, in a period when COVID-19 cases and deaths were minimal. Most restrictions implemented to help combat COVID-19 have been lifted; although strict hygiene and social distancing methods remained in place, life returned to some level of normality. However, global and local health authorities continued to use various media to inform the public about the epidemic and to promote a range of health protective behaviors to prevent infections [4,5]. In this in-between stage of the COVID-19 pandemic, our participants still perceived COVID-19 as a severe disease, and the recommended measures as highly efficient to prevent infection. This indicates a “danger control” process, in which individuals are motivated to take action to lessen the threat. Additionally, the “lack of fear control” and vulnerability scores indicated a strong reaction to the ongoing fear appeal communication about COVID-19, even if people did not consider themselves to be highly vulnerable [8].

### Comparisons With Prior Studies

Although individual protective measures were rather consensual in our study population, collective restrictions had more mixed acceptance rates—ranging from 80%-57%. One possible explanation is that these measures were assessed in light of their restrictive nature [31], socioeconomic consequences (eg, unemployment, bankruptcy of businesses, mobility restrictions), and/or psychological burden (eg, anxiety, depression) [32]. For instance, the stay-at-home order for nonessential workers was linked to health anxiety, financial worry, decreased physical activity, isolation, and loneliness [9,33]. Similarly, closing all educational settings (schools and universities) jeopardized students’ education and well-being [34-36], while closing bars and restaurants led to massive unemployment in the food and hospitality sector during the first lockdown. This would be in line with a European Union report documenting a substantial increase in people’s economic anxiety in the months following the COVID-19 outbreak, especially in those European Union countries hit hardest in economic terms [37], and with a survey conducted in the aftermath of the first quarantine periods showing that unemployment and poverty/social inequality were close behind COVID-19 in the global concerns ranking [38]. Conversely, isolating vulnerable people [39], forbidding mass gatherings, and restricting the mobility of nonessential workers had higher acceptance rates, as these targeted restrictions may reduce COVID-19 spread and deaths with more limited social costs.

The relationship observed between vulnerability and acceptance of collective and individual protective measures became nonsignificant when entered together with efficacy, lack of fear control, and perceived severity in the multivariate models. This indicates that the acceptance of collective restrictions was more related to collective than personal protection, likely to protect others [21] and restore the situation back to normal. The acceptance of collective restrictions was nevertheless lower among participants aged >60 years, who are more likely than others to be targeted and isolated from the rest of society [40]. Other indicators of vulnerability (chronic disease, perceived health) were unrelated to acceptance rates, perhaps because older age was the main identified factor linked to COVID-19 mortality during the first outbreak [41].

### Limitations

The results of this study must be viewed in light of its main limitations. First, the cross-sectional design does not allow causal inferences about relationships between variables to be determined. Furthermore, missing data precluded the investigation of EPPM appraisal in the total study sample, and some novel measures such as “location tracking” [19] or “COVID-19 passport” were omitted. Second, personality variables such as anxiety trait and pessimism may have a pivotal influence on appraisals and were not assessed. Finally, data were collected in a cohort including a small proportion of individuals with deprived socioeconomic backgrounds, which may limit the generalizability of our results. The large size of our cohort and the inclusion of diverse professions and socioeconomic groups nevertheless offered an interesting opportunity to assess the acceptance of COVID-19 nonpharmaceutical measures in the general population.

### Conclusion

The aim of this study was to evaluate the acceptance of COVID-19 nonpharmaceutical measures and, more specifically, to measure the public’s acceptance of these measures and their association with COVID-19 perceptions. Our findings suggest that acceptance rates of COVID-19 nonpharmaceutical measures were rather high, but varied according to their perceived social costs, and seemed to be more related to collective than personal protection. Altogether, it appears that the nonpharmaceutical measures that minimize social costs while controlling the spread of the disease are more likely to be accepted and therefore more sustainable during pandemics.

## Abbreviations

EPPM: Extended Parallel Process Model
RR: rate ratio

## Data Availability

The data underlying this article are available in Open Science Framework [42].
